# Enhancing hard X-ray beamline performance at SwissFEL through spontaneous radiation measurements

**DOI:** 10.1107/S1600577525010227

**Published:** 2026-01-01

**Authors:** Christoph Kittel, Masamitsu Aiba, Christopher Arrell, Ariana Cassar, Eugenio Ferrari, Nicole Hiller, Eduard Prat, Sven Reiche, Nicholas Sammut, Thomas Schietinger, Didier Voulot, Tobias Weilbach, Marco Calvi

**Affiliations:** a Paul Scherrer Institut Forschungsstrasse 111 5232Villigen PSI Switzerland; b University of Malta MSD2080Msida Malta; c CERN CH-1211Geneva 23 Switzerland; d Deutsches Elektronen-Synchrotron DESY 22603Hamburg Germany; RIKEN SPring-8 Center, Japan

**Keywords:** synchrotron radiation, free-electron laser, undulator, X-rays

## Abstract

The hard X-ray beamline (Aramis) of the Swiss free-electron laser (SwissFEL) has been in user operation since the end of 2017 and its performance has been continuously monitored and enhanced over the past eight years. From the beginning, spontaneous radiation has been utilized to monitor the behavior of its 13 individual undulator modules: the pointing direction of the electron beam in each module, their alignment relative to the electron beam, and the calibration of their magnetic field strength (*K* calibration). This article introduces the methods employed at the Aramis beamline to optimize performance using spontaneous radiation, traces the evolution of these improvements, and highlights the recently achieved record performance.

## Abstract 2

Methods using spontaneous radiation to monitor and optimize the Aramis hard X-ray beamline are presented, together with the eight-year evolution of these improvements and the resulting record performance.

## 1. Introduction

In an X-ray free-electron laser (FEL), a multi-GeV, high brightness and short pulse duration electron bunch with very low normalized emittance is injected into a long undulator line to generate very bright and short photon pulses in the X-ray wavelength range (Pellegrini *et al.*, 2016 ▸). SwissFEL, the Swiss free-electron laser at the Paul Scherrer Institute, uses a 6.1 GeV electron beam in the Aramis branch to generate photon energies up to 14 keV using a series of 13 in-vacuum, variable-gap undulator modules of 4 m length (Prat *et al.*, 2020 ▸; Milne *et al.*, 2017 ▸).

To achieve best performance, particularly in terms of photon pulse energy (of order millijoule), the electron beam trajectory must remain within a few micrometres of a straight line along the entire undulator beamline, with the undulator modules aligned to that line, and their magnetic field strengths calibrated. In this article, we present methods for fine-tuning both alignment and calibration using spontaneous radiation. After providing some basic information on our set-up in Section 2, we outline in Sections 3–8 the various measurement techniques employed, including both previously reported methods and novel approaches introduced here for the first time, such as fine pitch adjustment and the determination of the source point location for individual undulator modules. Section 9.1 reports historic data of the Aramis beamline performance over the years, highlighting the need for periodic component realignment and recalibration of undulator module strengths. In Section 9.2, we describe the newly refined beamline set-up procedure, which has enabled record performance levels for SwissFEL thanks to its enhanced accuracy and reproducibility, before concluding in Section 10.

## 2. Prerequisites

### 2.1. SwissFEL Aramis undulator line

The SwissFEL facility consists of a linear electron accelerator driving two undulator beamlines, a hard X-ray beamline called Aramis (Prat *et al.*, 2020 ▸), and a soft X-ray beamline called Athos (Prat *et al.*, 2023 ▸). In standard operation, a radiofrequency photoinjector generates electrons in bunches of 200 pC at 100 Hz repetition rate. These bunches are then accelerated by an S-band (3 GHz) booster to about 300 MeV and further, up to 3.3 GeV (Athos) or up to 6.1 GeV (Aramis), by a C-band (5.7 GHz) linac. Along the way the electron beam undergoes longitudinal compression in magnetic chicanes called bunch compressors.

The Aramis undulator line consists of 13 in-vacuum, variable-gap undulator modules, each 4 m long with a period length of 15 mm (Calvi *et al.*, 2018 ▸).

Operating with vertical gaps down to 3 mm to achieve magnetic field strengths of up to 1.28 T, the required relative wavelength accuracy of δλ/λ ≃ 10^−4^ calls for gap adjustments in steps as small as 0.3 µm. To compensate for the Earth’s magnetic field, a compensation coil of the dimension of the undulator module is mounted on the outside of the vacuum chamber and generates a vertical magnetic field to ensure that electrons wiggle around a straight trajectory.

Inserted between the undulator modules are several key components that ensure proper electron beam transport and phase matching of the radiation. Beam focusing is provided by quadrupoles, arranged in the classic FODO configuration. Each quadrupole is paired with a beam position monitor (BPM) (Stadler *et al.*, 2018 ▸) and equipped with correction coils. Their horizontal and vertical windings, co-wound with the main quadrupole coil, act as steering magnets and can be used, for example, in feedback loops to steer the beam to a specified transverse position as measured by a BPM. Each BPM-quadrupole unit is mounted on a common motorized support that can be remotely displaced both vertically and horizontally. This feature is essential for the electron beam based alignment (e-BBA) procedure, as described later in Section 2.3. Each intra-undulator section, between two undulator modules, further contains a phase shifter (a compact, tunable magnetic chicane) designed to delay the electron bunch by up to one full wavelength of the emitted radiation, thereby enabling phase matching between successive undulator modules, when the magnetic field strength of the undulator line changes.

Finally, after the last undulator module, the electron beam is deflected below ground into a specially designed absorber, known as the beam dump, while the photons are directed via a complex optical system consisting of mirrors, crystals, and focusing elements to one of three scientific instruments (endstations) (Follath *et al.*, 2016 ▸). The three endstations are called Alvra, Bernina and Cristallina, with Bernina being located on the straight path coming out of the undulator.

### 2.2. Photon instrumentation

**Figure 1:**
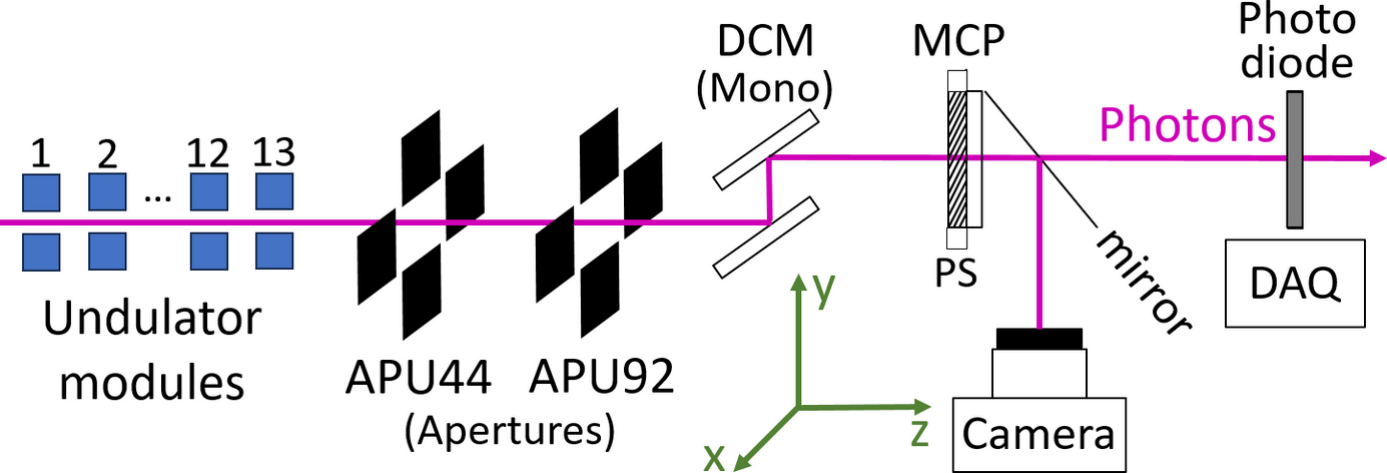
Sequence of photon beam instruments in the Aramis beamline and the Bernina endstation. From left to right: 13 undulator modules; variable-aperture slits (APU44 and APU92) for photon orbit definition; double-crystal monochromator (DCM) for wavelength selection; double-stage multi-channel plate (MCP) with a phosphorus screen (PS) and camera for profile monitoring; and silicon photodiode for direct intensity measurement, via a data acquisition system (DAQ) (used only during the commissioning period). All devices can be individually removed from the beamline to avoid obstructing the photon path.

The photon beam based approach detailed in this paper requires multiple instruments in the Aramis beamline and the Bernina endstation photon path. While the beamline and instrumentation is optimized for the high flux of FEL radiation, the components that are in use for the alignment are sensitive enough to detect the spontaneous undulator radiation. A schematic of the instrument sequence, including the axis definition, is shown in Fig. 1 ▸. All instruments can be inserted individually into the photon beamline as required. Two variable-aperture slits, APU44 and APU92, are located 44 m and 92 m downstream of the last undulator module, respectively. They primarily serve to define the beam trajectory for photon delivery to the experimental stations, but can also be used to assess the transverse source position (*x* and *y* offsets) of each module, as will be described later. A double-crystal silicon monochromator (Follath *et al.*, 2016 ▸; Ingold *et al.*, 2019 ▸) is used to select the photon energy of the undulator radiation with a relative bandwidth of about 10^−5^. Further downstream, two sensitive detectors can be inserted to measure very weak signals (on the order of nJ), to measure the spontaneous radiation of a single undulator module. The first is a double-stage multi-channel plate (MCP) detector, which is sensitive enough to capture the photon beam profile from spontaneous radiation in single-shot mode. Thanks to a high-speed camera, it can operate at the full repetition rate of 100 Hz. Additionally, a silicon diode is included in the set-up to measure total photon intensity when detailed profile information is not required. While the diode was used during early commissioning, the MCP became the primary diagnostic tool for regular operation, as it provides precise spatial information on the photon beam. In contrast, the diode’s measurement is subject to positional uncertainties, as its sensitive surface may not cover the entire photon beam.

### 2.3. Initial electron trajectory

To apply the advanced photonic techniques for optimizing FEL performance, detailed in the following sections, it is essential to first achieve a sufficiently straight electron beam orbit (<0.1 mrad) by applying an e-BBA. This step is essential to ensure that the spontaneous emission from all undulator modules can be reliably observed on a common detector located more than 100 m downstream of the final module and to provide a reasonable starting point for further detailed optimization.

The most established e-BBA procedure is the dispersion-free steering method, originally introduced for the commissioning of the Linear Coherent Light Source (Emma *et al.*, 1999 ▸) and later refined and adapted to other facilities (Kang & Loos, 2019 ▸; Scholz *et al.*, 2019 ▸; Ferrari *et al.*, 2023 ▸). While it provides the highest precision by virtue of its capacity to correct localized orbit distortions within the undulator line, it requires measurements at vastly different beam energies, rendering its implementation both complex and time-consuming.

Instead, we adopt a faster (on the order of minutes) and more practical approach, which comes with the downside of lower accuracy and thus necessitates further optimization. The approach exploits the high degree of symmetry exhibited by the FODO lattice of the Aramis beamline and the assumption of a systematic kick common to all undulator modules. This method involves calibrating the magnetic axes of the quadrupoles and steering the beam through their centers while simultaneously minimizing the spread of all corrector currents (Aiba & Böge, 2012 ▸).

As we will see later in Section 4, the electron trajectory determined by e-BBA may be further optimized by means of spontaneous radiation (photon beam based alignment or p-BBA). Furthermore, it may turn out that the electron trajectory found by optimizing corrector currents and FEL output results in a photon beam that is not exactly centered on the target experimental station. In such cases, a global adjustment of the electron trajectory will still be needed, typically achieved by shifting the entire undulator line, while keeping the trajectory feedback running, to realize the necessary changes in slope and offset of the trajectory (on the order of a few microradians or micrometres, respectively).

## 3. Spontaneous undulator radiation

The use of spontaneous radiation to optimize the performance of an FEL was first proposed by Tanaka (Tanaka *et al.*, 2012 ▸) during the commissioning of SACLA (Ishikawa *et al.*, 2012 ▸). Unlike lasing, spontaneous radiation is emitted independently by each undulator module, making it a valuable diagnostic tool. Its wavelength is given by the resonance condition, 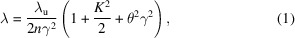
where λ_u_ is the period length of the undulator, γ the Lorentz factor of the electron beam, *n* the harmonic number, *K* the undulator deflection parameter and θ the observer angle with respect to the undulator axis (corresponding to θ = 0). The dimensionless deflection parameter *K* describes the magnetic field strength and corresponds to the maximum deviation angle of the electrons in the horizontal plane, divided by the Lorentz factor. It can be expressed as 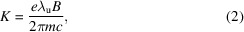
where *B* is the magnetic field amplitude, *e* and *m* are the charge and the mass of the electron, respectively, and *c* is the speed of light. The wavelength is related to the photon energy, *E*_γ_, through the Planck relation, *E*_γ_ = *hc*/λ, where *h* is the Planck constant.

**Figure 2:**
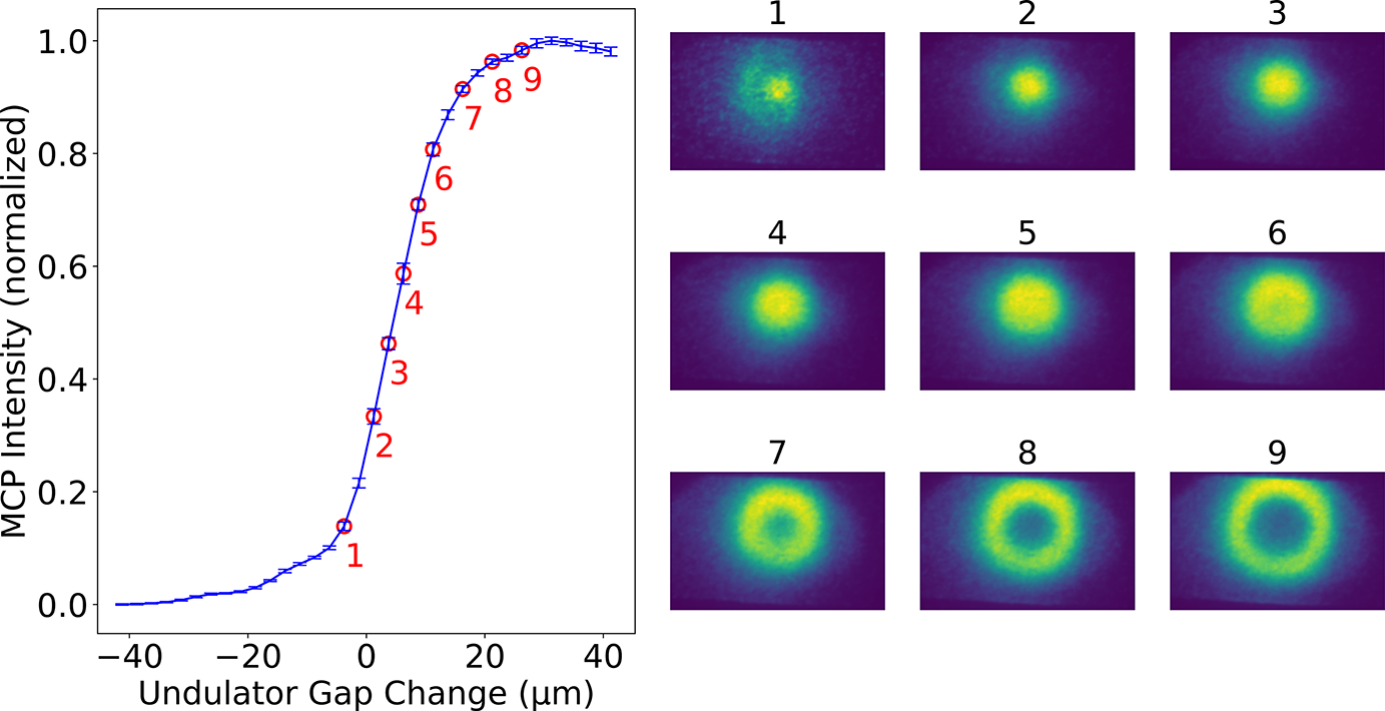
MCP intensity versus undulator gap scan at 10.8 keV photon energy (left). Red numbers indicate the gap positions corresponding to the MCP images shown on the right (averaged over 25 shots). The image sequence illustrates the evolution of the transverse profile during the gap scan around a *K* value of 1.4. The reference frame is arbitrarily set around an initial guess of the gap.

In Fig. 2 ▸ we give an example of a spontaneous undulator radiation measurement, as it is generally used for the alignment and calibration of single undulator modules (see Sections 6 and 8). All measurements of this kind require a photon energy filter, *i.e.* a monochromator, to enable selective scanning of the energy of the spontaneous radiation cone. In the example shown, the gap of a single undulator module is scanned around a predicted value from previous calibrations (*e.g.* simulations, magnetic measurements or photon calibrations) for a fixed monochromator energy of 10.8 keV to vary the magnetic field strength. The figure shows the monochromated transverse profile of the spontaneous radiation at various gap sizes. As the gap opens, the integrated intensity detected by the MCP begins to increase when the gap approaches the resonance condition, rising sharply to a maximum before decreasing again, defined as the gap at which the maximum intensity is reached at the center of the spot (θ = 0). The rising side is commonly referred to as the *blue edge*, as it corresponds to the high-photon-energy side when the undulator module gap is fixed and the wavelength filtered by the monochromator is varied. The decreasing side (low energy) will be called the *redshift* later in the text. Observation of the beam profile reveals that, as the undulator module moves away from resonance in the center of the spot by increasing the gap, the resonance and consequently the intensity shifts to larger angles (θ), forming a hollow, doughnut-shaped beam.

The quality of this type of spontaneous radiation measurements strongly depends on the energy spread (σ_E_) of the electron beam. During our gap scans, we typically work with an electron beam of a reduced energy spread of σ_E_ ≃ 1.5 × 10^−4^. This is achieved by a weaker compression setting, resulting in a bunch length of around 200 fs (compared with the regular 20 fs).

When measuring spontaneous radiation of a single undulator module, it is important to suppress the signal of the other modules along the line. This is done by detuning the *K* values of the other undulator modules and adjusting the monochromator to the resonance photon energy of the module to be measured. When detuning the *K* values of the other modules, it is necessary to shift them sufficiently to prevent them from generating significant background on the MCP. Increasing their *K* values is generally preferable, as this lowers the harmonic photon energy and avoids the strong background on the redshift side, thereby allowing smaller detuning steps, which in turn keeps close to lasing conditions (*e.g.* natural undulator focusing, wakefields).

## 4. Electron trajectory refinement by photon beam based alignment

After the e-BBA, trajectory deviations from a straight line are small but can be further minimized by steering the photon beams generated by individual undulator modules to a specified center position on the MCP, thereby leveraging the long distance to the detector. This trajectory fine-tuning typically begins with one undulator module, where upstream and downstream BPM readings are used for alignment. The process is facilitated by a trajectory feedback system, which adjusts corrector magnet currents depending on BPM target positions. The latter are varied until the photon beam reaches the specified position on the MCP.

In principle, this alignment procedure could be started with any undulator module. For practical reasons, however, it is advantageous to start with the central unit (module number 7 in our case) and thus divide the alignment process into two halves, which are performed backwards and forwards. This approach reduces the error accumulated from module to module (mainly due to the angular resolution of the pBBA) by roughly a factor of two compared with one single alignment. More importantly, it also reduces the maximum adjustments required to correct systematic errors left over from the eBBA (*e.g.* a slightly curved orbit), which may exceed the available correction range if the alignment process is started from the first or last module. For this central module only, we divide the trajectory change equally, but with opposite signs, between the entrance and exit BPMs, to maintain the initial offset at the center of the module. We proceed in the downstream direction by adjusting the exit BPM target position (downstream of each module) and in upstream direction by adjusting the entrance BPM target position (upstream of each module). So for each module, except for the central one, only one BPM target is adjusted, ensuring that the trajectory in previously aligned modules remains unaffected.

This procedure has two effects: it further improves the straightness of the electron trajectory, as we have confirmed empirically, and it optimizes the MCP data quality for subsequent steps in the alignment procedure.

In analogy to the e-BBA, the method is commonly referred to as photon beam based alignment, or p-BBA for short.

The following section introduces a quantitative method for evaluating the trajectory straightness by determining the photon source location of each undulator module.

## 5. Photon source location

**Figure 3:**
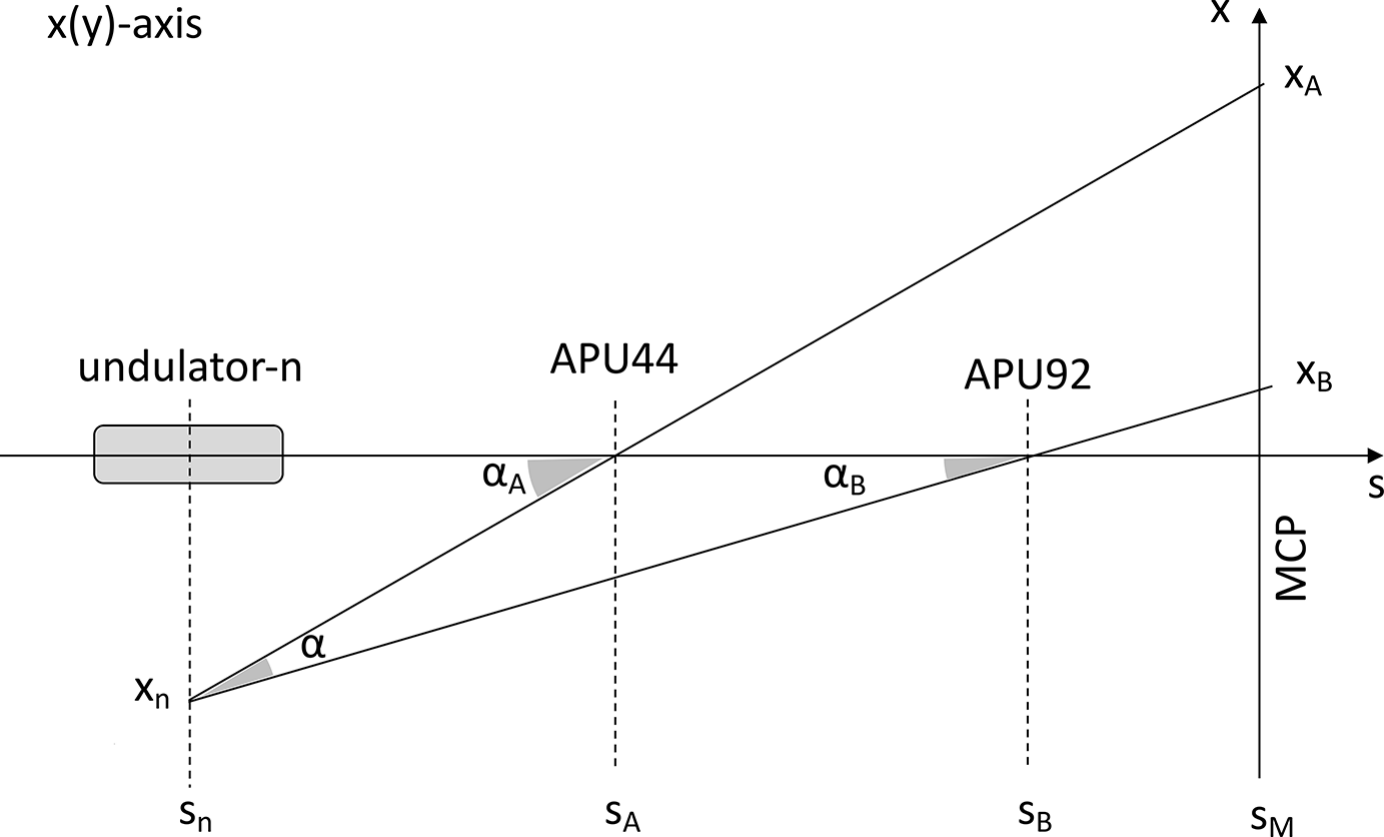
Schematic of the source point triangulation concept (horizontal plane): the undulator module source is imaged twice on the MCP screen, first with slit APU44 (A) closed and later with slit APU92 (B) closed. Similar considerations can be applied to the vertical plane.

The two slit systems described in Section 2.2 can be used to determine the source point of an undulator module’s spontaneous radiation. The concept of this source point triangulation is illustrated in Fig. 3 ▸. In essence, the beam position on the MCP is measured for two situations: first after narrowing (down to about 0.1 mm) the upstream slit, then again after closing the downstream slit accordingly. Assuming the undulator module to be a point-like source, its position can be determined from simple geometric relations, as shown in the figure. The reference frame of the MCP may slightly differ from that of the slits owing to assembly tolerances; however, this discrepancy does not affect the measurement, as only the relative difference between the two spot positions is relevant for alignment purposes, as explained in the following. What is important is the reproducibility of their center position, which is known to be better than 0.1 µm, due to their absolute encoders.

To simplify the calculations, we apply the paraxial approximation, which allows the trigonometric functions to be evaluated in the small-angle approximation, where 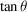 ≃ θ. The relevant formulas are as follows, 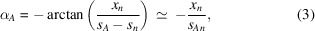
where α_*A*_ is the incident angle of the radiation at the first slit, *x*_*n*_ is the horizontal displacement, *s*_*A*_ is the position of the first slit, *s*_*n*_ is the position of the undulator module, and *s*_*An*_ = *s*_*A*_ − *s*_*n*_ is the distance between the first slit and the undulator module. Analogously, for the second slit we have
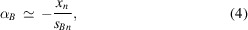
where α_*B*_ is the incident angle at the second slit, and *s*_*Bn*_ = *s*_*B*_ − *s*_*n*_ is the distance between the second slit and the undulator module. The angular distance α between the two slits as seen from the undulator module is given by 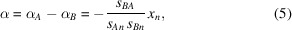
where *s*_*BA*_ = *s*_*B*_ − *s*_*A*_ is the distance between the two slits. The horizontal distance between the two spots on the MCP screen, *x*_*A*_ − *x*_*B*_, is related to α and can be expressed as
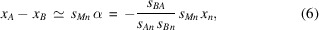
where *s*_*Mn*_ = *s*_*M*_ − *s*_*n*_ is the distance between the MCP (*s*_*M*_) and the undulator module. To further simplify the relationship, we introduce a proportionality constant 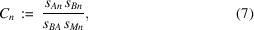
which allows us to express the undulator module source horizontal displacement *x*_*n*_ in terms of the measured spot distance *x*_*AB*_ = *x*_*A*_ − *x*_*B*_ on the MCP screen, 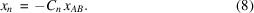
For the first and the last (13th) undulator module along the beamline, *C* is calculated to be 1.93 and 0.8, respectively. A value of *C* < 1 is preferable, as it magnifies the undulator module position error on the MCP screen. Thus, in principle the best measurement sensitivity is achieved with the first slit located as close as possible to the source (undulator module) and the second slit positioned directly in front of the MCP screen.

**Figure 4:**
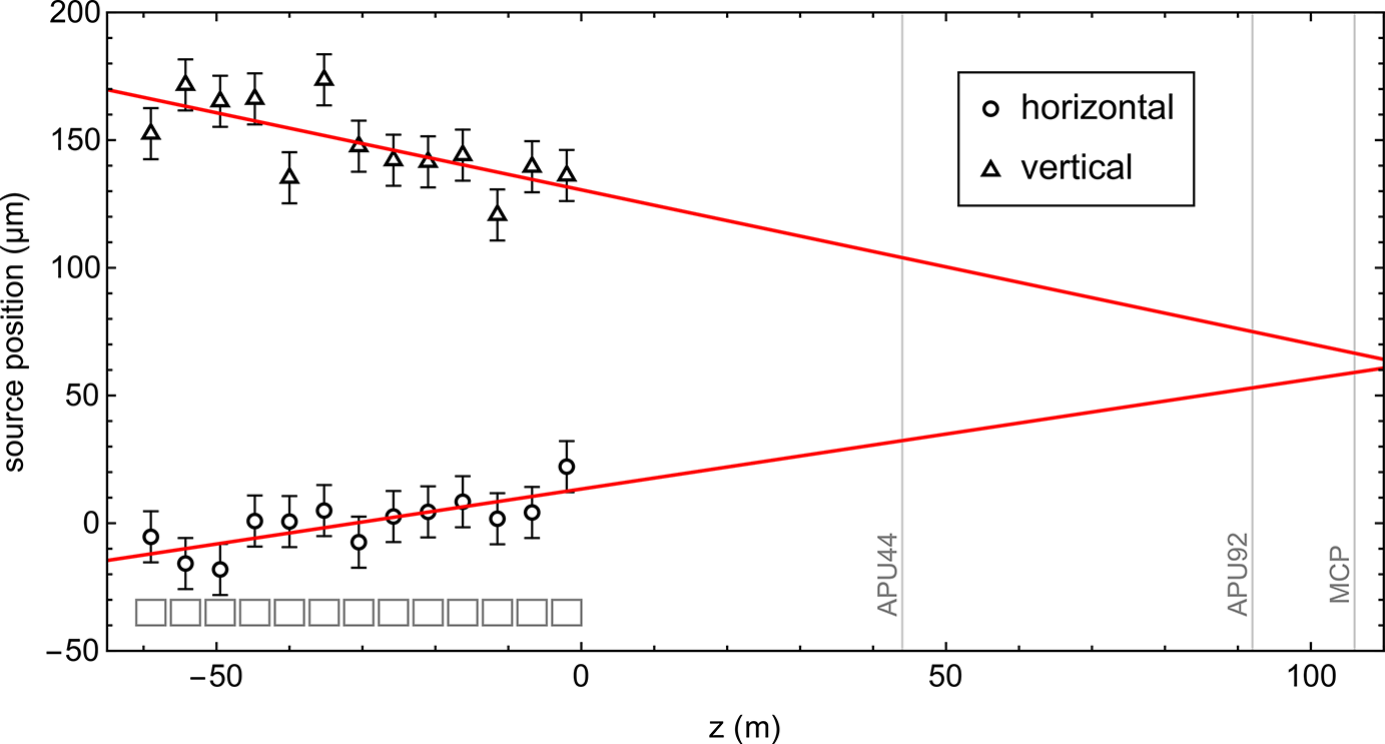
Source point analysis of the undulator modules along the Aramis beamline applying the described method. The horizontal and vertical positions are shown with an associated uncertainty of ±10 µm, and their linear fits are superimposed as solid red lines. The *z* positions of the undulator modules are indicated by gray rectangular boxes, the locations of the apertures and the MCP are marked by vertical lines.

Ideally, once the offset is estimated, the beam trajectory within the module should be adjusted accordingly, and the measurement should be repeated to verify whether the two spots overlap. The results of the analysis, shown in Fig. 4 ▸, indicate that occasional discrepancies between the slit alignment and the photon beam pointing may occur. While this does not invalidate the source point method, it does call for more detailed analysis. In such cases, only deviations from a straight trajectory need to be considered to optimize the lasing signal.

We conducted a series of measurements to validate the source point method and estimate its accuracy. Specifically, known offsets were artificially introduced via the trajectory feedback target values for the BPMs in a selected undulator module and subsequently estimated using the present method. This confirmed the validity of the approach and yielded an uncertainty of approximately ±10 µm, primarily due to the granularity of the MCP profile. The latter depends on the 20 µm diameter of its channels, whose combined signals form the measured beam profile. Although this level of precision does not meet the stringent tolerances required for FEL fine-tuning (in our case estimated to be within 1 µm), the method remains useful for identifying significant misalignment, for instance following extended maintenance periods during which components may have been physically displaced, realigned, or otherwise affected by construction activities.

## 6. Undulator alignment: height and pitch

Once the electron beam trajectory along the undulator line is sufficiently straight, the height and pitch of each undulator module can be fine-tuned to align their magnetic centers within ±10 µm with this trajectory, while the good field region (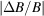 < 10^−4^) in the *y* direction is about 100 µm. For this adjustment, we exploit the variation of the undulator module strength around its magnetic center in the transverse horizontal (*x*) and vertical (*y*) directions. For the planar undulator modules used in Aramis, the strength parameter *K* increases parabolically with displacement in the *y* direction, while it remains constant in the *x* direction, because of the width of the magnetic pole tips (15 mm).

**Figure 5:**
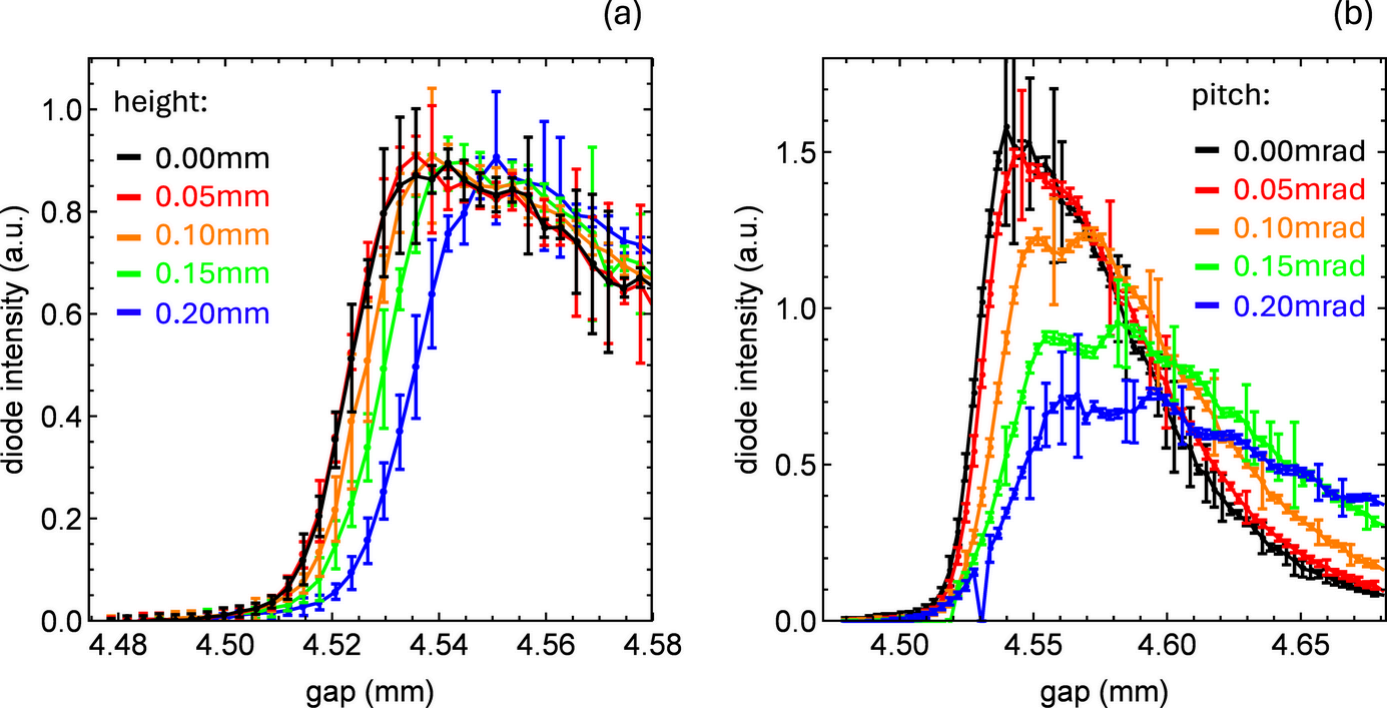
Gap scans at fixed photon energy set by the monochromator performed (*a*) at different heights and (*b*) at different pitches. The zero height and zero pitch correspond to the undulator module center, which was determined prior to this measurement. Negative values are omitted for clarity, as they are identical to their positive counterparts due to the field’s symmetry about the center.

Fig. 5 ▸(*a*) illustrates this approach with gap scans performed at different heights, where 0 indicates the aligned position, demonstrating the shift of the harmonic position, marked by the maximum radiation intensity, toward larger gaps when moving off-center in *y*. We measured both positive and negative height and pitch positions, but, for clarity, show only the positive positions, since the negative positions yield equivalent results due to the field’s symmetry about the center axis.

**Figure 6:**
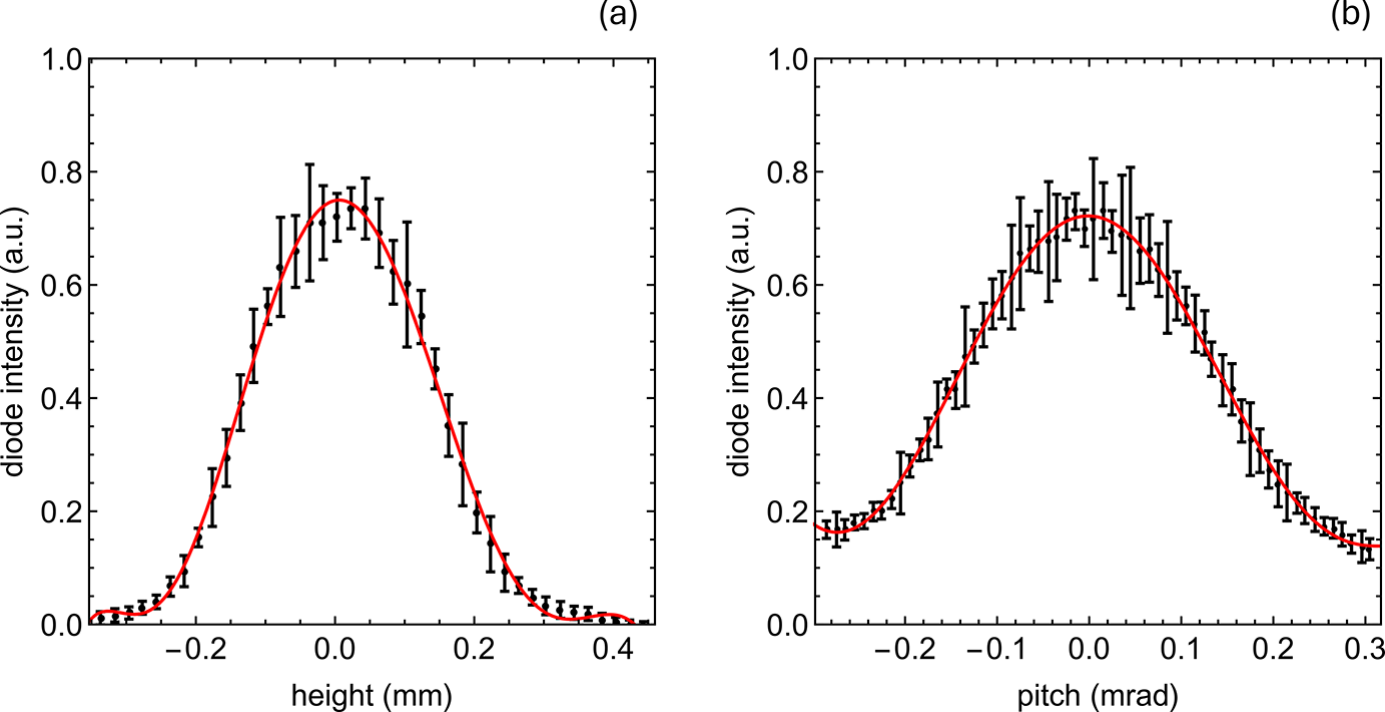
(*a*) Height and (*b*) pitch scans at fixed photon energy set by the monochromator and a fixed gap, which was selected to lie on the blue-edge side of the spectrum.

A more efficient approach is based on performing the measurements on the blue edge of the harmonic and directly scanning the height or the pitch, while searching for maximum signal intensity. This procedure provides a rather precise estimate of the magnetic center, as shown in Fig. 6 ▸; for clarity, height = pitch = 0 is already the aligned state.

The gap scans for different pitch values, as seen in Fig. 5 ▸(*b*), do not exhibit a steady shift of the harmonic, as is the case for height changes, where only minor focusing effects distort the resonance. Instead, significant pitch errors reduce the slope of the blue edge and its maximum intensity, while also generating oscillations on the redshift side.

Both effects can be explained by the varying magnetic field strength experienced by the electron beam along the undulator module. The field can be well approximated by the following expression of its main Fourier component, originally derived for pure permanent magnet undulators, 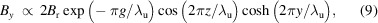
where *B*_r_ is the remanent field of the magnets. This formula conveniently estimates the deviation upon vertical displacement from the axis (*y*), which in good approximation results in *K* ∝ 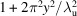. It allows a quick estimate of the relative variation of *K*: for a change of 10^−4^, the corresponding vertical displacement is about 34 µm, while for 10^−3^ it is about 105 µm. The latter effect is directly visible in the gap scans (Fig. 5 ▸), whereas achieving the former requires performing a height scan as shown in Fig. 6 ▸. This procedure allows for reliable alignment along the entire undulator axis, ensuring that FEL amplification is not compromised.

In conclusion, while height errors could also be effectively compensated by adjusting the undulator module strength (via gap adjustment), pitch errors will significantly degrade FEL performance. This underlines the crucial importance of remote control of both height and pitch, as is standard practice for all undulator modules in SwissFEL.

## 7. Earth magnetic field compensation

**Figure 7:**
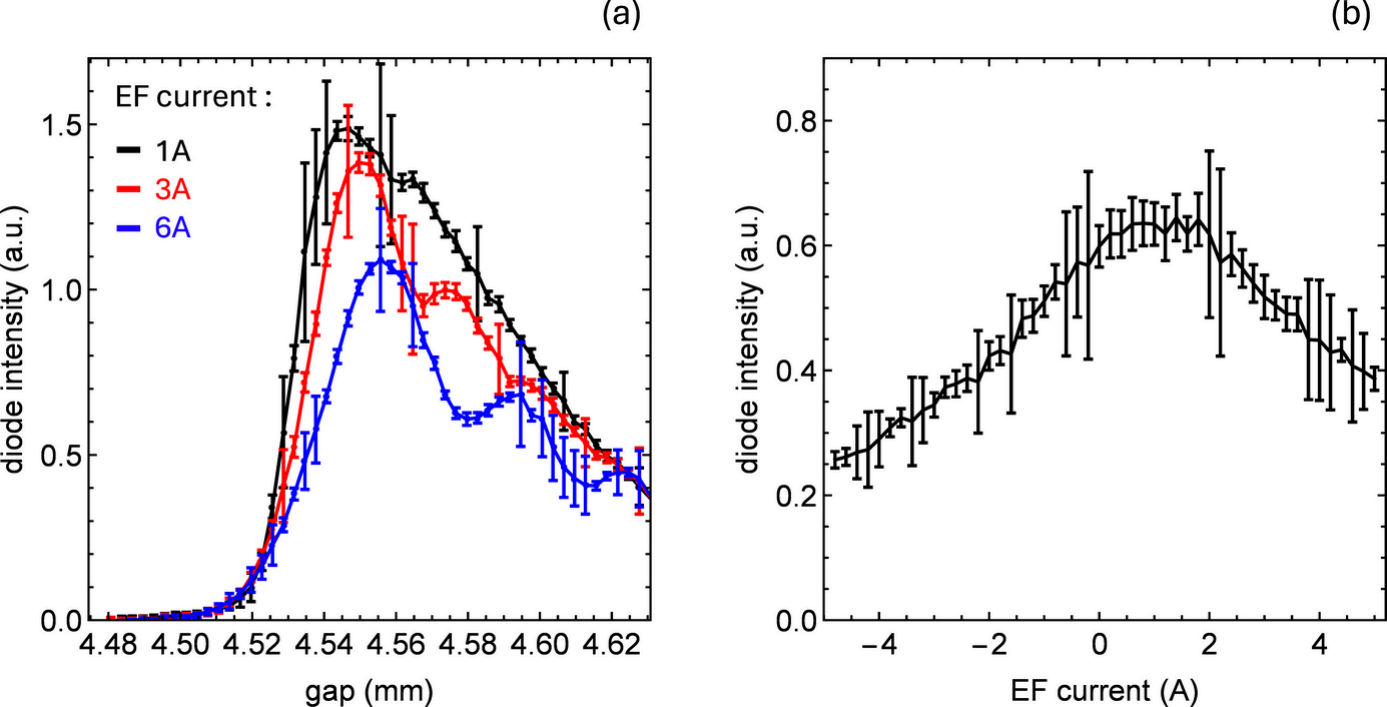
(*a*) Gap scans at a fixed photon energy set by the monochromator, performed for different currents in the Earth field coil. (*b*) Intensity as a function of current in the Earth field coil at a fixed gap corresponding to the blue edge.

For a typical planar undulator with linear horizontal polarization, the surrounding magnetic field needs to be compensated in good approximation only in the vertical direction (Calvi *et al.*, 2018 ▸). Thus, for the Aramis undulator modules, only coils to counter the vertical magnetic field component were adopted, called Earth magnetic field compensation coils, or Earth field coils for short, since the effect is dominated by the Earth’s magnetic field. The coils exhibit a transfer function of 190 µT mA^−1^ between current and field integral. Fig. 7 ▸(*a*) shows the photon flux intensity recorded after the monochromator for various coil currents. The changes in harmonic shape resemble those observed for changes in pitch; in this case, however, the reduced slope and oscillations are attributed to the overall bending of the electron trajectory along the undulator module. This bending superimposes the wiggling motion and diminishes the FEL amplification effect. On the red-shifted side, the local maxima indicate harmonic splitting, as observed at different angles by the detector.

The photon intensities shown in Fig. 7 ▸(*b*) were measured at a fixed gap corresponding to a position along the blue-edge slope, as a function of the coil current. The data confirm the optimum correction strength of about 200 µT m, which is consistent with the estimates made during the magnetic characterization of the devices prior to installation. In the range 0 to 2 A there are practically no measurable effects, suggesting a blind region for spontaneous radiation, where bending does not significantly impact harmonic quality, while FEL suppression occurs much earlier because of the smaller opening angle of the FEL beam with respect to that of spontaneous radiation, θ 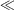 (λ/*L*)^1/2^.

## 8. Gap versus *K* calibration

After the geometrical alignment of all undulator modules the systematic optimization of the FEL output at a certain photon energy necessitates accurate control of every undulator module’s magnetic strength, quantified by its *K* value. In practice this calls for an accurate calibration between the set undulator gaps (read back with linear encoders) and the resulting *K* values. To ensure consistency between the calibrations of different undulator modules, it is essential to maintain the electron beam energy at a constant value throughout the entire calibration measurement campaign. (If this is not possible for some reason, a reference undulator module may be used.) For absolute accuracy of the *K* values within a tolerance of δ*K*, the electron beam energy must be known to a corresponding accuracy of δ*E* given by 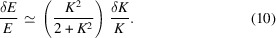
For our nominal operating range between *K* = 1.2 and *K* = 1.8 the conversion coefficient is approximately in the range 0.5 ± 0.1. Once the beam energy is known, the Lorentz factor of the electrons is computed as γ = *E*/*m*_e_ with *m*_e_ = 511 keV, which, in conjunction with the photon energy of the calibrated monochromator, allows the determination of the corresponding *K* value.

In the Aramis undulator line we perform the calibration procedure for the usual *K* range between 1.2 and 1.8. To improve fitting accuracy, we define a measurement set of *K* values with intervals of 0.1, derived from the resonance condition [equation (1)]. These sets of *K* values and the corresponding photon energies are used to scan the gap of each undulator module around the value predicted by the previous calibration (during the first commissioning we used the calibration established by magnetic laboratory measurements). In practice, the monochromator is set at the photon energy corresponding to the given *K* value, and the intensity curve is measured by scanning the undulator gap around the predicted value. It is preferable to scan the undulator gap rather than the monochromator to determine its optimum value, as this directly results in a gap setting corresponding to the *K* value in question. This procedure is then repeated for the entire set of *K* values and photon energies, and for all undulator modules.

**Figure 8:**
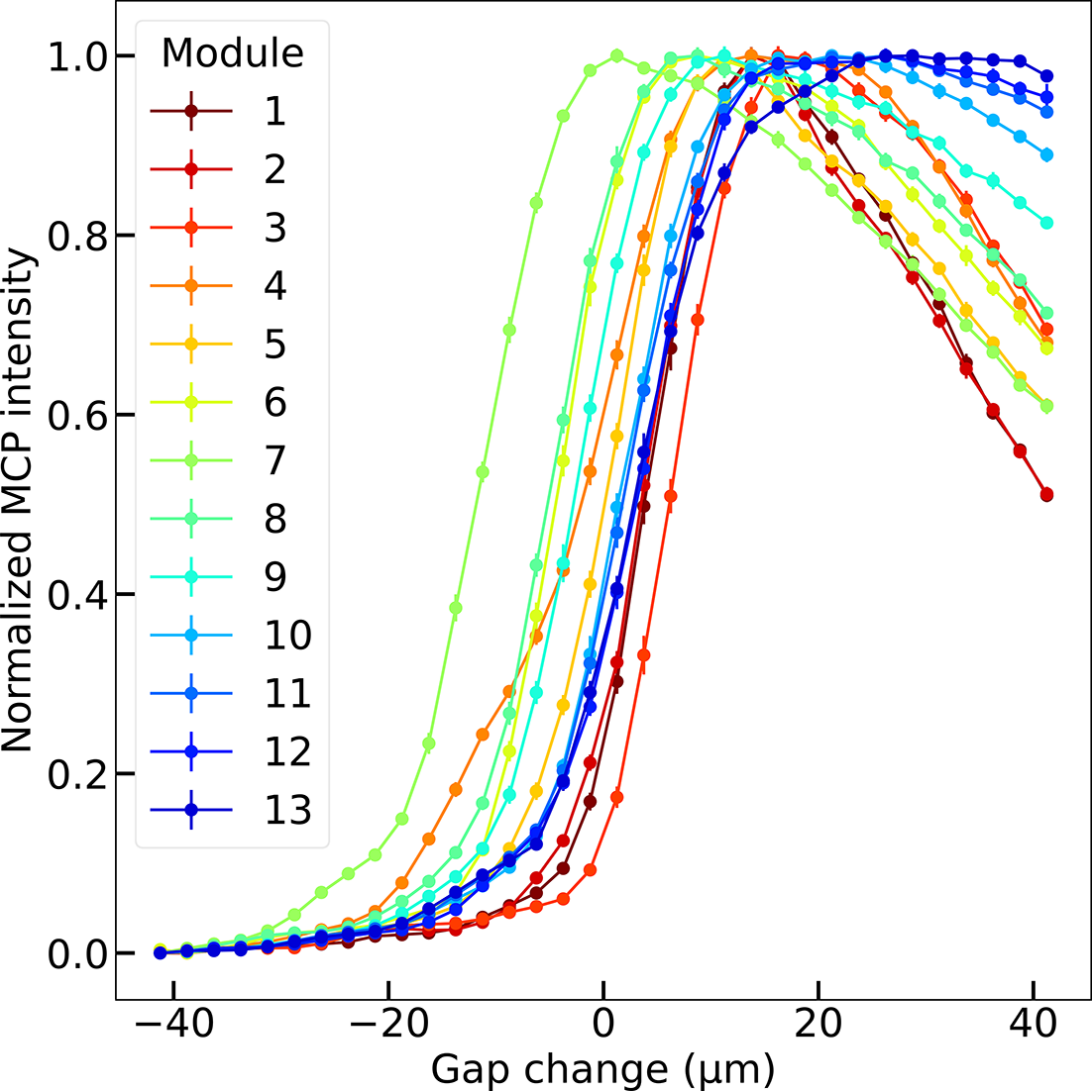
Example of *K* calibration scans at a specific monochromator photon energy of 10.06 keV (*i.e.* a single *K* value of 1.5) for all undulator modules of the Aramis line recorded in November 2024.

**Figure 9:**
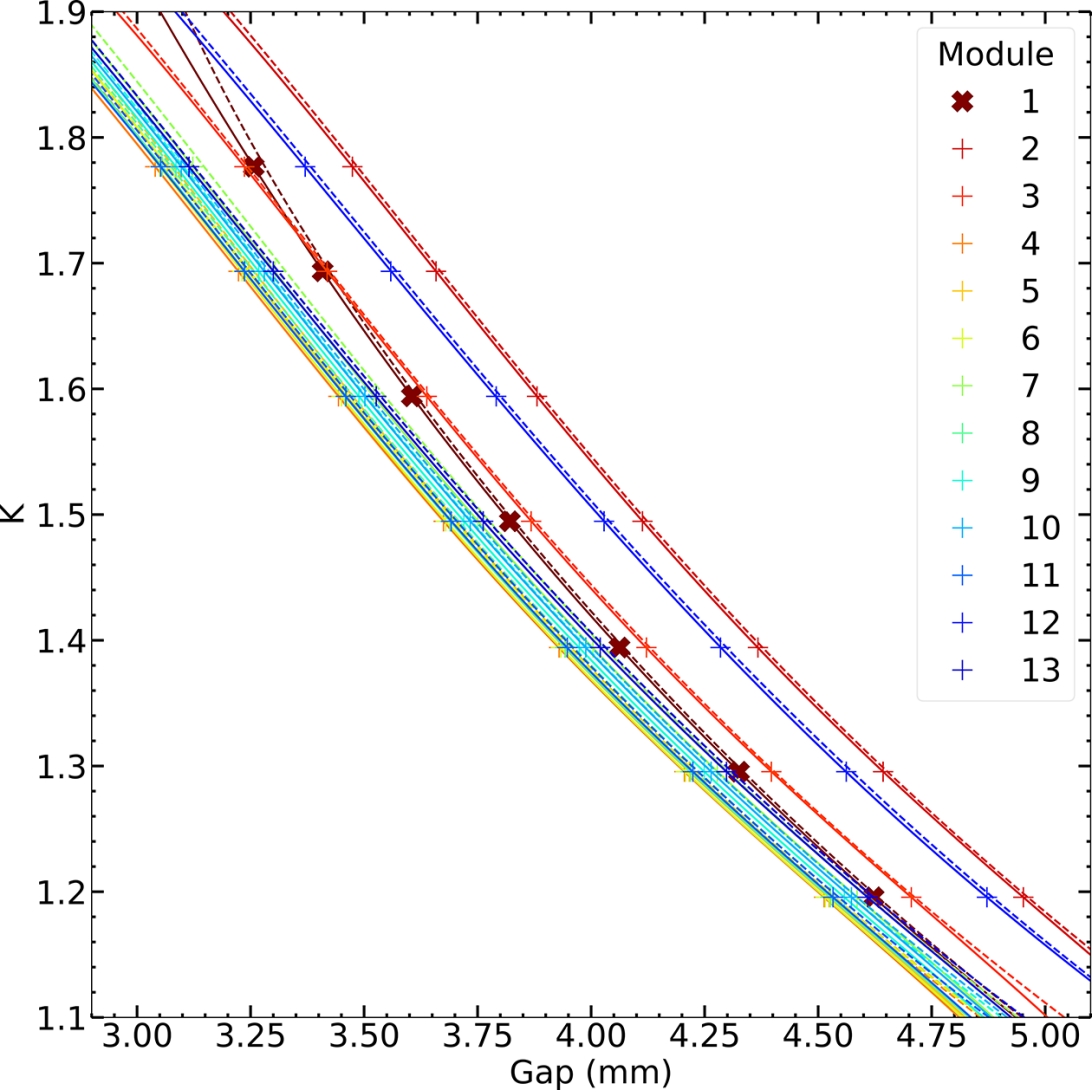
Final result of the *K* calibration procedure carried out in November 2024. The plot shows the *K* values (plus signs) as a function of the undulator gap (center of the error function fit) for each undulator module. For the interpolation a fourth-order polynomial fit is used. Solid lines mark the latest calibration fits, dashed lines show the previous calibration fits for comparison.

During the most recent measurement campaign in November 2024, we repeated the gap scans for all 13 undulator modules. The electron beam energy was kept constant at 5.8 GeV for all measurements. Fig. 8 ▸ shows gap scans for all modules around *K* = 1.5. The curves are fitted with an error function to determine the gap value corresponding to the blue edge. The fit function is *f*(*x*) = *a*_1_ + *a*_2_{erf[*a*_3_(*x* − *x*_0_)] + 1}, a simplified version of the function defined by Tanaka *et al.* (2012 ▸), where erf is the error function and the fit parameter *x*_0_ corresponds to the center of the edge. Fig. 9 ▸ summarizes the result of the *K* calibration procedure, namely the relation between the *K* value (computed from the monochromator photon energy) and the corresponding undulator gap (found by the blue-edge fit) for all 13 modules. For these curves, a fourth-order polynomial is used to fit the data, which is later used by the control system to compute the gap for a desired *K* value.

The significant differences between modules can be largely corrected by applying simple gap offsets, which account for assembly tolerances in these large devices and suboptimal mechanical calibration. This applies to all modules except the first, which deviates from the regular behavior due to its somewhat distinct magnetic design: it served as the prototype for optimizing the series production, and is highlighted in Fig. 9 ▸.

Fig. 9 ▸ not only shows the result of the most recent calibration campaign, but also, for comparison, the previously applied calibration.

**Figure 10:**
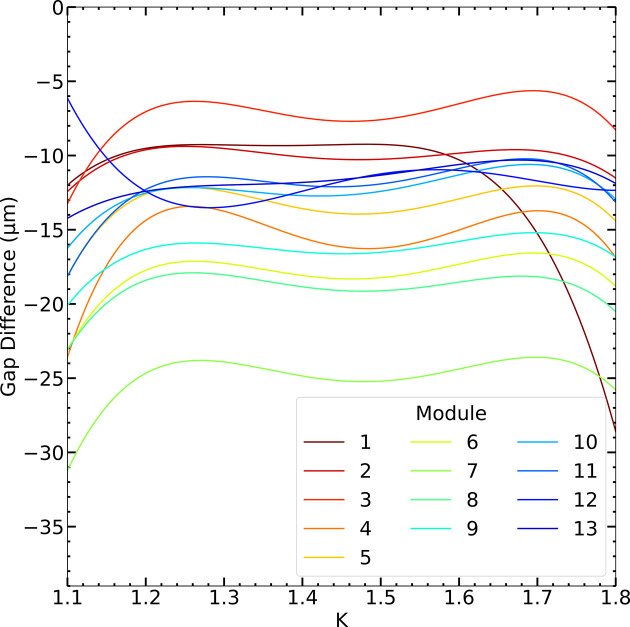
Differences between the newly measured calibration fits (undulator gap to *K*) and the previous ones for the 13 individual modules over the measured *K* range. The systematic shift towards smaller gaps may be attributed to the electron beam energy calibration.

For better visibility we show in Fig. 10 ▸ the differences between new and old calibration curves in terms of gap adjustments for the 13 modules. The calibration change remains roughly constant throughout the *K* range, with differences of up to 18 µm between individual modules. The fact that all gap differences are negative is most likely due to a slightly different calibration of the electron beam energy in the newly measured data. Only for the first module are the changes more pronounced at *K* values above 1.65. Although the difference in overall shape can be attributed to its distinct magnetic design, it remains unclear why it also exhibits rather large deviations between the previous calibration and the most recent one.

Since 2016, four undulator calibration datasets (*K* versus gap of all undulator modules) have been established, allowing us to track the beamline’s evolution in terms of undulator settings, and to better understand the uncertainties inherent in the applied technology. Although the undulator module system is designed for sub-micrometre (<0.3 µm) fine tuning, reproducibility has proven problematic. First, mechanical hysteresis leads to variations in the *K* values depending on whether the gap is closing or opening, a limitation that can be mitigated. Second, radiation damage can cause an inhomogeneous change in magnetization along single undulator modules (Bizen *et al.*, 2016 ▸). Third, temperature changes pose a problem, not only because of the known effects on permanent magnets (Clarke, 2004 ▸) but also because of mechanical expansion, which, due to the complexity of the system, does not fully return to its original state after one cycle. Temperature changes of 1–2 K can occur during extended shutdowns with tunnel access and, as experience shows, during activities involving the installation of new heat-generating hardware, *e.g.* gas attenuator vacuum pumps near the undulator line. Even these small temperature variations are sufficient to induce measurable effects, ultimately degrading performance. Although the encoders give consistent readings, the magnetic field can still deviate from its calibrated state by up to 0.1%, exceeding tolerances and requiring recalibration. This happens because the encoders do not measure the magnetic gap directly. Instead, they record the positions of the girders, where the magnetic arrays are mounted, at two points along a 4 m length outside the vacuum chamber. From this, we conclude two things: first, the mechanical elements between the encoders and the gap are not perfectly rigid; second, the gap profile is not always a straight line, as confirmed by magnetic measurements.

## 9. Operational experience at SwissFEL Aramis

We complement our discussion on undulator alignment and optimization with a brief overview of the performance evolution of the SwissFEL Aramis line (Section 9.1), followed by an account of a recent optimization campaign incorporating the previously discussed methods and findings (Section 9.2).

### 9.1. Performance evolution

**Figure 11:**
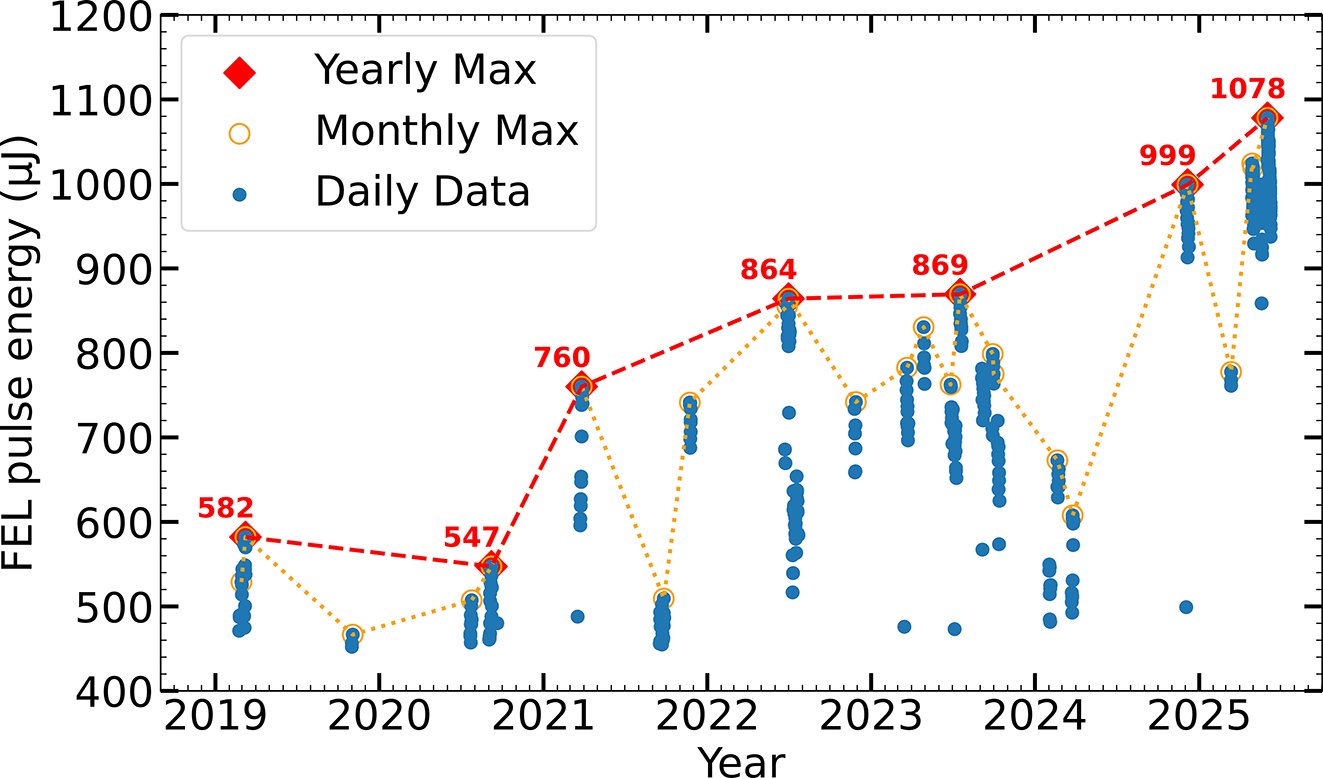
Performance evolution of SwissFEL over the past years. The pulse energies for photon energies between 11.8 and 12.4 keV are shown, where a straight electron trajectory and precise alignment and calibration of undulator modules are of particular importance.

Fig. 11 ▸ traces the performance evolution of Aramis around 12 keV over the course of the last seven years (Hiller *et al.*, 2025 ▸). Following initial commissioning at 12 keV in 2019, SwissFEL achieved pulse energies of approximately 550 µJ, enabled by a comprehensive undulator calibration and alignment campaign (Schmidt *et al.*, 2019 ▸). By 2021, maximum pulse energies increased to 760 µJ, primarily due to a reduction in slice energy spread of the electron beam. This was realized by decreasing the longitudinal dispersion (*R*_56_) in the laser heater chicane, allowing for enhanced compression (Prat *et al.*, 2022 ▸).

In 2022, further improvements in trajectory alignment within the undulator line yielded pulse energies near 860 µJ (Ferrari *et al.*, 2023 ▸). While this performance was sustained with considerable operational effort (in particular empirical optimization) until mid-2023, a gradual degradation was observed thereafter, with pulse energies declining to approximately 600 µJ by mid-2024. The degradation was attributed to trajectory and undulator misalignment, as either the first or the last four undulator modules did not appear to contribute to the FEL output. Frequent target adjustments and trajectory optimizations were considered the likely cause.

In November 2024, several days were allocated to a comprehensive campaign for realigning and recalibrating both the trajectory and undulators. As a result, a new facility record was achieved, with pulse energies exceeding 1 mJ at 12 keV photon energy (see Fig. 1 ▸). The procedure applied during this campaign is described in detail in the following section.

Our experience underlines the importance of a carefully designed, systematic realignment and recalibration procedure that takes into consideration all aspects of the undulator system and that must be repeated at regular intervals.

### 9.2. Most recent optimization campaign

In November 2024, we applied, for the first time, most of the previously described alignment and calibration steps in one sequence, followed by a thorough optimization of the FEL output at 12 keV photon energy. In the following we summarize the various steps of the full set-up and optimization procedure and present the result of this performance enhancement effort.

The first step in the procedure is the determination of an initial electron beam trajectory. For this we apply the fast e-BBA described in Section 2.3, in which the trajectory requiring the minimum corrector strengths through the undulator line is identified. While this procedure improves orbit straightness, it does not necessarily align the FEL beam with the beamline optics.

Therefore, the next step consists of verifying the FEL pointing by observing the photon beam. The beam is sent through the two apertures described in Section 2.2 and observed on a downstream scintillating (YAG) screen. The observation of a clipping effect when closing the apertures to the nominal FEL beam size indicates a global misalignment of the electron beam with respect to the X-ray optics, which is corrected by adjusting the position and angle of the entire sequence of quadrupole magnets within the undulator as one rigid system.

Following the pointing adjustment, a p-BBA as described in Section 4 is carried out to further refine and straighten the electron trajectory, thereby establishing the reference orbit for the final optimization.

Once the electron trajectory is established, each undulator module is aligned to it by performing height and pitch scans, see Section 6. With this procedure, we ensure that each module is in a position and orientation where its magnetic axis aligns with the electron trajectory.

With the certainty that all undulator modules are aligned to the FEL propagation direction, the next and last step of the initial undulator set-up consists of the calibration of the undulator module strengths (*K* calibration), according to the procedure described in Section 8.

**Figure 12:**
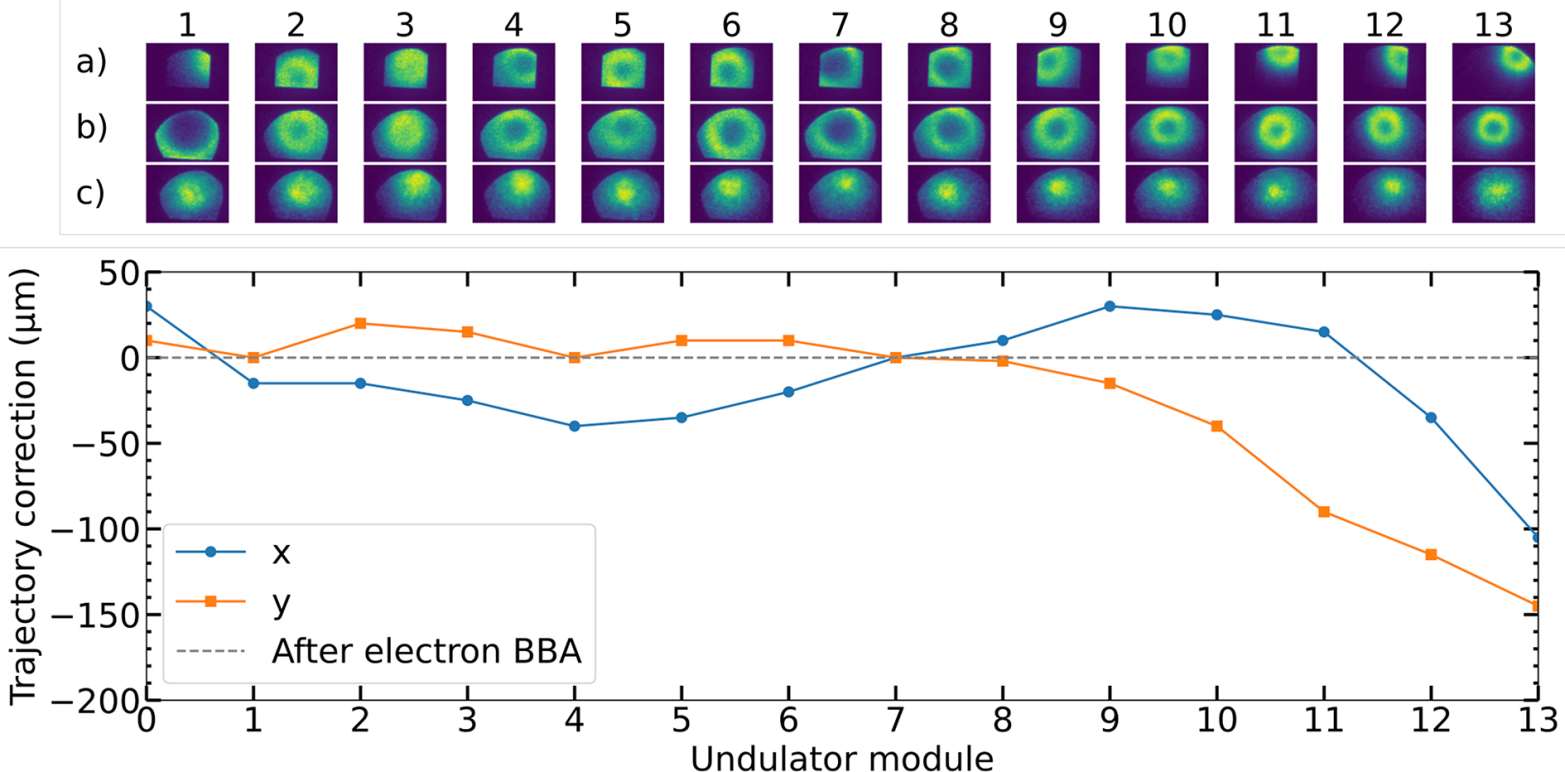
Top: spatial distribution of undulator radiation (indicating FEL alignment and *K* calibration) from each of the 13 undulator modules as seen by the MCP downstream: (*a*) after the e-BBA, (*b*) after the subsequent p-BBA, and (*c*) after application of the *K* calibration. Bottom: changes in electron trajectory between e-BBA and p-BBA.

Fig. 12 ▸ illustrates the various steps of the undulator alignment and calibration procedure as it was executed in November 2024. The top part of the figure shows MCP images of the spontaneous radiation emitted by individual modules, first after the e-BBA (*a*), then after centering the propagation axes of the spontaneous radiation via p-BBA (*b*), and finally after adjusting the undulator strengths to the same value (*K* calibration) (*c*). The bottom part of the figure displays the changes in electron trajectory as a result of the p-BBA, which ensures that all undulator modules emit spontaneous radiation in the same direction, as shown by the center row of the MCP images shown above.

The systematic set-up of electron trajectory, undulator alignment and calibration is followed by a more empirical optimization or fine-tuning of various parameters affecting the electron beam and its interaction with the undulator field. First, the electron beam quality is optimized with regard to maximum FEL output, starting from the photoinjector electron source. Here, maintaining a low electron beam emittance is essential for achieving high FEL performance. Our target for the normalized projected emittance is 250 nm in both transverse planes, measured after the first two S-band accelerating structures (at 150 MeV) with a well matched beam optics (Prat *et al.*, 2019 ▸).

Key parameters for the low-energy beam optimization are the gradient of the gun accelerating field, the strength of the gun solenoid magnet, but also the settings of quadrupole corrector magnets built into the gun solenoid. Other important factors are the size and the quality of the transverse profile of the photocathode laser, which often requires adjustments, in particular after downtimes or interventions involving the laser system. Of critical importance is proper alignment of the laser beam through the UV capillary, which is used to clean the transverse mode of the UV laser pulse after frequency doubling.

The initial bunch compression set-up ensures a peak current at the core of the bunch of about 3 kA after the two bunch compressors. [This value is significantly higher than the one reported by Prat *et al.* (2020 ▸), since at that time the energy spread of the beam at the injector was larger, requiring less compression for optimal performance.]

For stable FEL performance, minimal electron beam dispersion throughout the undulator is essential. We therefore measure the dispersion and correct for it. The most common source for dispersion is an error in the assumed beam energy. The dispersion measurement thus suggests a correction for the beam energy, which is used to scale all quadrupole strengths accordingly. If required, residual dispersion is compensated with dedicated corrector quadrupoles in the dispersive regions of a chicane in front of the undulator (so-called energy collimator).

Finally, the electron beam optics require empirical fine-tuning, for matching the quadrupoles preceding the undulator section. For the adjustment of the electron beam tilt we can generally perform systematic corrections, but also here, in most cases, fine-tuning of the quadrupoles, skew quadrupoles and sextupoles located in the chicanes is sufficient. The optimization is based on the observed FEL pulse energy and therefore already requires a satisfactory performance level of the FEL. It may later be repeated to reach ultimate performance.

The high-quality electron beam is then used to fine-tune the undulator module parameters, starting from their initial settings given by the systematic set-up. In particular we begin with all undulator modules at the same (calibrated) *K* value. We then apply undulator tapering to ensure effective energy exchange between the electron beam and the radiation field all along the undulator (Kroll *et al.*, 1981 ▸; Jiao *et al.*, 2012 ▸). Specifically, we apply a linear taper throughout the undulator beamline to compensate for energy loss due to wakefields and a quadratic postsaturation taper, both optimized with respect to FEL output power.

Next, the phase shifters between undulator modules (see Section 2.1) are adjusted to ensure constructive interference between modules and maximize overall coherence. At this stage it is important to verify that all undulator modules make a significant contribution to the FEL process. We accomplish this by detuning the modules one by one, while observing the corresponding reduction in FEL pulse energy.

A further refinement of the electron trajectory, on top of e-BBA and p-BBA, is provided by the so-called adaptive orbit correction (Gaio & Lonza, 2015 ▸). This approach correlates single-shot trajectory data with the simultaneously recorded FEL pulse energy (beam-synchronous data acquisition) and keeps track of the trajectories resulting in the highest pulse energies. The target positions for the trajectory feedback are then updated accordingly to bring the orbit to the new optimum. A priori, the method relies on natural trajectory fluctuations, which may turn out to be too small to produce significant fluctuations in pulse energy. In this case, it may be helpful to add random fluctuations to the trajectory feedback target positions to increase the search range and improve optimization robustness. Since the p-BBA is typically performed using a low-energy-spread beam, residual trajectory shifts of a few tens of micrometres can still occur when switching to a compressed beam, due to shifts in its center of mass. Adaptive orbit correction has proven to be an extremely fast and efficient optimization method. However, it is important to ensure that all modules contribute to the FEL performance, otherwise the algorithm can easily end up in a local optimum.

The general method of correlating single-shot machine sensor data with simultaneously recorded FEL output to optimize target values for the corresponding feedback may be applied to other feedback loops as well. In fact we have successfully applied the method to the feedback loops controlling the compression settings.

Finally, the *K* values along the undulator line are empirically re-optimized by scanning the strength of each undulator module individually.

The empirical optimization steps described above are typically repeated over several iterations to converge on the maximum FEL performance.

The first application of the above-described procedure to the SwissFEL Aramis beamline in November 2024 resulted in a new record FEL pulse energy of 1.03 mJ at 12 keV photon energy. In the meantime, the pulse energy at 12 keV has been further pushed to 1.08 mJ.

## 10. Conclusions

We have described the use of spontaneous radiation measurements at the SwissFEL Aramis beamline both to improve our understanding of the undulator system and to enhance overall performance of the FEL. In particular, we have shown how the spontaneous radiation of individual undulator modules can not only be used to align and calibrate these modules but can also play a crucial role in refining the electron trajectory previously found using conventional (electron) BBA techniques.

To illustrate the practical relevance of our results, we have included an account of a recent campaign to align, calibrate, and optimize the Aramis undulator at SwissFEL. It systematically incorporated all relevant measurements and adjustments based on spontaneous radiation, in conjunction with more general optimization steps concerning the electron beam. The result was a new record performance of this beamline, exceeding 1 mJ FEL pulse energy at a photon energy of 12 keV. More importantly, the applied procedure has proven to be robust and well reproducible under regular operating conditions.

Future work on spontaneous radiation measurements will focus on, among other things, improving the photon source location method, as it shows great promise to become a fast and versatile tool for verifying and correcting the electron beam trajectory.

On the FEL side, the excellent performance reached at 12 keV encourages us to explore lasing with the Aramis line beyond its nominal photon energy range, *i.e.* at photon energies up to 15–16 keV. On a longer timescale, we aim at even higher FEL performance throughout the accessible photon energy range by further improving the electron beam quality, in particular by reducing the intrinsic energy spread.
